# MRI of the Colon in the Pharmaceutical Field: The Future before us

**DOI:** 10.3390/pharmaceutics11040146

**Published:** 2019-03-27

**Authors:** Sarah Sulaiman, Luca Marciani

**Affiliations:** Nottingham Digestive Diseases Centre and National Institute for Health Research (NIHR) Nottingham Biomedical Research Centre, Nottingham University Hospitals NHS Trust and University of Nottingham, Nottingham NG7 2UH, UK; msxss56@nottingham.ac.uk

**Keywords:** magnetic resonance imaging, MRI, large intestine, gut, large bowel, volume, transit, motility, flow

## Abstract

Oral solid drug formulation is the most common route for administration and it is vital to increase knowledge of the gastrointestinal physiological environment to understand dissolution and absorption processes and to develop reliable biorelevant in vitro tools. In particular, colon targeted drug formulations have raised the attention of pharmaceutical scientists because of the great potential of colonic drug delivery. However, the distal bowel is still a relatively understudied part of the gastrointestinal tract. Recently, magnetic resonance imaging (MRI) has been gaining an emerging role in studying the colon. This article provides a comprehensive; contemporary review of the literature on luminal MRI of the colonic environment of the last 15 years with specific focus on colon physiological dimensions; motility; chyme and fluids; transit and luminal flow. The work reviewed provides novel physiological insight that will have a profound impact on our understanding of the colonic environment for drug delivery and absorption and will ultimately help to raise the in vitro/in vivo relevance of computer simulations and bench models.

## 1. Introduction

Oral solid formulations are the most popular way of drug manufacturing [1] and their dissolution in the gastrointestinal (GI) environment is a determinant process of drug absorption [2]. Therefore, it is key to improve understanding of GI dissolution processes as well as the factors that affect them to enhance the efficiency of oral pharmaceutical forms. These factors are related to drug properties (e.g., dose, pKa, solubility, diffusion coefficient, crystal form, permeability, particle size) and also to the gastrointestinal conditions as well such as anatomical and physiological (liquid and non-liquid contents, buffer capacity, pH, bile salts, flow, motility, transit, and membrane) characteristics [2,3]. It is known that these characteristics continuously change throughout the GI tract, with some becoming less favorable more distally except for pH, which generally rises [4].

Current animal models as well as in vitro and in silico biorelevant models predict inadequately drug bioavailability because there are still “uncharted waters” in the field of drug absorption in the GI tract [5,6]. Since the majority of the new drugs approved are delivered per os there is a need of creating reliable predictive in vitro tools that not only reflect the complexity of the in vivo conditions but that can also be applied throughout the whole manufacturing procedure of oral formulations [7].

The latest approach on improving drug manufacturing procedures and performance includes designing and using biorelevant in vitro predictive techniques based on data acquired from in vivo measurements. These techniques are believed to be the future for assessing drug absorption because they are expected to cover the needs for a toolkit that prevents inaccurate discarding of drugs in the preclinical phase, reduces the use of animals and human subjects and offers realistic evaluation of new, old, and controlled dosage forms during their development and quality control. To fulfil all these requirements, the toolkit has to reflect the diversity and the complexity of the in vivo conditions [1,2]. Currently, despite all the previously developed biorelevant models there still exists a huge gap, and therefore a potential, between the in vitro and in vivo correlations [3]. When it comes to oral modified release formulations, the design of one representative predictive model that combines all the determining in vivo conditions becomes even more challenging, due to the lack of knowledge of the lower GI tract physiology. Formulations evaluation may remain inaccurate because of the compromises that are made when existing tools are used [1,2,3]. It is proposed that the enhanced evaluation of modified release formulations is going to derive from a toolkit that is going to reflect the in vivo conditions that determine drug release [2].

The colon forms the distal part of the human gastrointestinal tract [4]. Colonic length and diameter are estimated to be around 150 cm and 5 cm respectively [5]. Its absorbing surface area is about 0.05 m^2^ and there are no villi. The anatomical regions from proximal to distal consist of the cecum, the ascending colon, the transverse colon, the descending colon and the sigmoid colon [5]. As the rest of the GI tract, it is formed by an inner mucosal layer, where absorption and secretion occur, the submucosal layer where nerves, lymphatics, and connective tissue are located, a smooth muscle layer divided in the longitudinal and circular muscle and the outer serosal area [4]. Mucus is formed in two layers in the colon and the inner one is 50–200 μm thick and strongly attached to the epithelium compared to the outer one which is easily removable [6].

The colon’s primary function is to ferment food components and absorb water, vitamins and electrolytes [4] whilst transforming the discarded material into faeces. Only little secretory activity occurs [7]. Colon secretion concerns mostly potassium, water and bicarbonate [7]. Nerves exert a significant role in controlling the colonic environment. Colonic activity is determined by the enteric nervous system (ENS) which controls the smooth muscle and the mucosa, has autonomy, and is responsible for transmitting colonic information to the rest of the body with its sensory and motor neurons. Apart from the ENS, the colon is regulated by external nerves that belong to the parasympathetic and sympathetic system and that are transmitters, too. Abnormal motor regulation of the colon can also happen on abnormal epithelial permeability due to injuries, inflammation and modified gene expression [4]. It naturally hosts the biggest part of human microbiota and therefore has a huge role in the immune system and pathophysiology mostly by balancing host and microbiota interaction with the mucosal barrier [8]. The GI tract, colon included, is a rather complex area to study because of its multiple role (irregular motility, movement of the contents, and secretion) that interplay with each other [9]. Understanding colonic physiology is pivotal for drug development because of its role in both functional and non-functional diseases [8,9]. MRI is an attractive tool to explore the colonic environment because of the absence of invasiveness and therefore the more biorelevant insights that it can provide on undisturbed colonic physiology and pathology. In healthy and diseased subjects, MRI is applied for the evaluation of gut motility, transit, and dislocation of its contents (liquid, non-liquid, gaseous) which can be quantified during the same study session, thereby reducing the number of possible appointments required and providing added value [9].

Pharmaceutical formulations that deliver the integrated drugs specifically to the site of the colon are considered not only for the treatment of colonic diseases but also for alternative ways of delivering active substances. The latter originates from the low enzymic and proteolytic activity of the site, which allows the intact absorption of proteins and peptides, the higher local residence times and the increased effectiveness of absorption enhancers in the large intestine. It is important though that the colon-targeting oral formulations can protect the active substances until they reach the colon for the delivery to occur and therefore it is vital to gain a better understanding of the physiology of the GI tract [4,8]. Conditions in the colon differ significantly from the rest of the GI tract and colon-targeted oral forms should be designed and tested on tools that reflect in vivo conditions, e.g., colon residence time which can be more than 24 h. In the early phase of drug assessment, colon-targeted drug formulations should be developed also using in vitro and in silico techniques designed on relevant in vivo data, and for this to happen deeper knowledge of colon physiology is needed. These tools could provide enhanced accuracy and reduce costs. So far, the applied in vitro tests are conventional and exclude important characteristics such as colon volumes and hydrodynamics [1,10].

Whilst knowledge of the upper GI tract is improving, the lower GI tract remains much less explored, partly because of the difficulty of access under physiological, undisturbed (unprepared) conditions [7]. Imaging techniques have the potential to enrich the current knowledge by providing new non-invasive insights on the undisturbed GI tract, with the real-time assessment of the physiological environment surrounding drug products until they reach the blood or lymphatic stream [6,9].

The most common imaging techniques in the medical field are based on X-rays (e.g., computed tomography), ultrasound and radionuclides (gamma scintigraphy), endoscopy, and magnetic resonance imaging (MRI) [6]. Computed tomography and gamma scintigraphy provide a radiation dose, which is undesirable and endoscopy is invasive and requires unphysiological bowel preparation [6,10,11]. The application of ultrasound techniques in the in vivo assessment of oral dosage forms is not common because of the small field of view and the adverse effect of the presence of gas on ultrasound waves propagation [6]. MRI has been emerging as a unique alternative to the limitations of the imaging methods mentioned above [9] and has been gaining increasing attention in the pharmaceutical field [11,12,13,14,15,16,17,18,19,20]. MRI is non-invasive and based on radio frequency waves, therefore it does not provide an ionizing radiation dose. It offers good image quality with excellent soft tissue contrast. Multiple parameters can be acquired during the same study day using a variety of techniques (for example cine, dynamic, tagging). MRI also carries limitations, such as the unsuitability for subjects who may have metal implants such as cardiac pacemakers or infusion pumps [11], has a high cost of instrumentation (though the cost of a scan is limited compared to other invasive techniques), it is motion-sensitive and carries a burden of data processing [6,12,19,21,22,23,24,25,26].

MRI has been applied for the study of functional GI diseases (irritable bowel syndrome, functional dyspepsia) and also other bowel diseases such as Crohn’s, scleroderma and colorectal cancer. One limitation is the need to standardize the methods and compare against gold standards [1]. More specifically, MRI has been used as an additional tool to assess the impact of mesalazine administration in the treatment of diarrhoea-predominant irritable bowel syndrome by quantifying the small bowel water content and using these data as an indicator for intestinal tone [12]. In the case of Crohn’s disease, MRI has been suggested as a tool for the follow-up of the disease while antibody to tumour necrosis factor (anti-TNF) is administered to patients [13,14]. MRI has been validated against endoscopy with good correlation between the two techniques and is considered to be reliable to assess the effect of anti-TNF on the disease state [14]. MRI has been also considered and tested in colon cancer assessment [15,16,17]. MRI application in staging of advanced colorectal cancer was evaluated against histopathological staging and it was found to be reliable in assessing T3-T4 colorectal cancer [15]. MRI has gained a role also in the assessment of liver and hepatic metastases from colorectal cancer as well as for the colon mucosal layers, including the potential for colon cancer staging [16,17].

Over the past 15 years, MRI has become an emerging tool for pharmaceutical sciences and it is timely to review its contribution to investigate the most unexplored area of the gastrointestinal tract. This review is a comprehensive summary of MRI of colon function in terms of dimensions, chyme and fluid characteristics, motility, transit and flow, and it highlights the application of MRI in understanding these key factors of the large intestine that determine oral drug bioavailability.

## 2. Colon Anatomy and Physical Dimensions

The data on colon organ geometric, physical dimension is scarce and mostly based on prepared bowel or post-mortem measurements [18]. MRI is uniquely suited to measure body organ three-dimensional shape and dimensions. These investigations can be very helpful to understand better the space to which drug products are delivered.

The segmental diameters of the colon were measured in 12 healthy volunteers as part of a study assessing the effects of administration of senna tea and erythromycin on the gut environment [19]. The minimum ascending colon diameter in response to senna tea and erythromycin was 3.48 cm and 3.4 cm respectively whereas the maximum one was 3.83 cm and 3.74 cm respectively. In the segment of the transverse colon the minimum values were 3.38 cm and 3.31 cm while the maximum ones were 3.49 cm and 3.48 cm respectively. Lastly, the minimum respective results for the descending colon were 2.83 cm and 2.77 cm while the maximum ones were 2.93 cm and 2.89 cm respectively.

Food effects can change the volume of this organ substantially. Ascending and transverse colon diameters were also measured by MRI in 16 healthy subjects who were fed 40 g of either glucose, fructose or inulin diluted in 500 mL of water or a mixture of 40 g of glucose and 40 g of fructose [20]. Fructose is malabsorbed in the bowel and was expected to increase fluid inflow to the colon; inulin ferments in the colon and was predicted to increase colonic gas volume. Indeed, colonic gas rose up to higher levels (as assessed by calculating the AUC from the volume versus time plots, yielding mean (95% CI) values of 33 (20) L·min) on inulin consumption rather than glucose and glucose-fructose scheme (both *p* < 0.05). At t = 255 min after fructose administration compared to glucose caused a larger diameter increase in the transverse colon (30% (43%) and 8% (21%) respectively). This effect was more evident in this segment rather than in the ascending colon where the biggest change took place at t = 75 min (18% (20%) and 4% (26%) respectively).

The role of lactulose ingestion (10 g diluted in 200 mL of water) on the intestinal environment was evaluated in healthy participants (*n* = 16) and patients with irritable bowel syndrome (IBS, *n* = 52 in total) in fasted and fed conditions [21]. During the study, MRI was used to assess the diameter of the large intestine as well. Overall, this study concluded that lactulose decreased the diameter of the ascending colon possibly due to the dysfunctional ileocecal region of IBS patients. Lactulose ingestion decreased significantly the ascending and transverse colon diameter of the diarrhoea predominant IBS (IBS-D) subjects compared to the healthy ones (*p* = 0.020 and 0.045 respectively). At the same time, it caused a distension in the descending colon of all groups (all IBS *p* = 0.014, IBS-D *p* = 0.03, constipation predominant IBS or IBS-C *p* = 0.006 and healthy *p* = 0.014) except for the mixed IBS or IBS-M subgroup where it reduced its diameter (*p* = 0.043).

**Figure 1:**
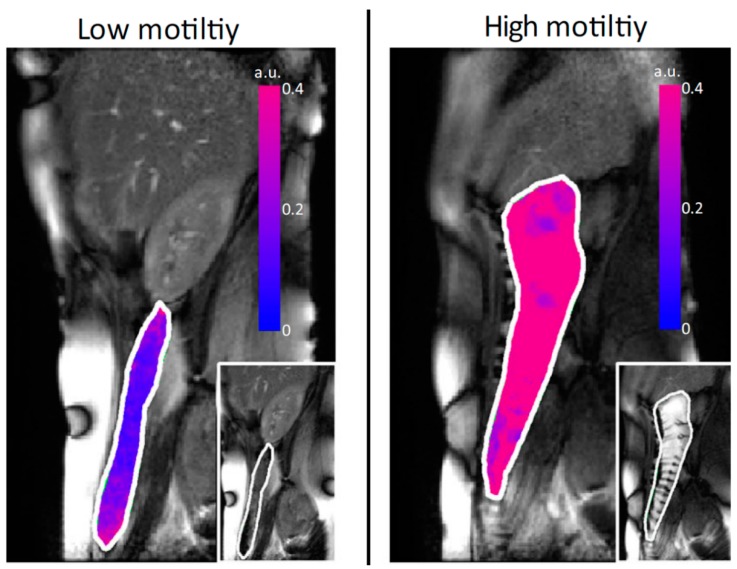
Motility assessment by the cine MRI technique. The figure depicts overlay (red/blue) of standard deviation of Jacobian. Reproduced from [28], Blackwell Publishing LTD, 2015 with permission.

A different study estimated the length of the lower intestine and rectum [22] using a novel tracking system (Motilis Medica SA, Lausanne, Switzerland) which consists of electromagnetic capsules that can be detected while they travel through the gastrointestinal tract and compared this to the findings from MRI scanning, which could not be performed at the same time. The length of the whole organ was assessed in 25 healthy subjects. The length was 95 (75–153) cm as measured by the electromagnetic capsule tracking system method and 99 (77–147) cm as measured by MRI, *p* = 0.15 (CV% = 7.8%). The MRI-measured length of the cecum/ascending colon was found 26% (*p* = 0.002) smaller than for the capsules method possibly to higher retention times of the capsules in this segment but this was the only significant difference between the two techniques (all *p* > 0.05). More specifically, MRI assessed the length of ascending, transverse, descending and rectosigmoid colon as 16 ± 23.5 cm, 27.8 ± 5.4 cm, 23.7 ± 4.1 cm, and 27.8 ± 11.2 cm respectively and electromagnetic capsule findings were 22.0 ± 7.5 cm, 28.4 ± 4.7 cm, 24.0 ± 7.4 cm, and 24.7 ± 8.7 cm respectively. Figure 1 displays an image of the whole abdomen for colonic length determination acquired by MRI.

## 3. Colonic Motility

Motility in the colon is erratic. Inter-individual and intra-individual variability can also affect the residence times in each segment of the colon, which in turn could influence oral drug bioavailability. Applications of MRI to study the colonic motility patterns are recent and expanding. MRI has the advantage of requiring no unphysiological bowel cleansing, which is instead required for manometric studies.

The bowel motility response to senna tea and erythromycin were measured in 12 healthy subjects by identifying the intestinal diameter of the various segments [19]. Senna generated a significant rise in the peristalsis of the ascending and descending colon. Specifically, senna caused, on average, 71.6% significant modifications in the ascending colon, 80% in the transverse colon, and 55% in the descending colon whereas erythromycin caused 60%, 46.67%, and 60% respectively. The mean difference of the percentage of the significant modifications was 13.31%.

The intestinal motion activity was also studied by the application of multi-nuclear ^19^F and ^1^H MRI in two healthy subjects who were administered one ^19^F capsule on study day 1 and two others with 300 mL of water on study day 2 and each time a meal and a commercial fibre drink was used for bowel distension [23]. On study day 1, the investigators identified high local bowel motion. The estimated forward velocity for subject A was 0.27 mm/s and for subject B 0.38 mm/s and the mean capsule velocity was 1.0 mm/s and 1.0 mm/s respectively for a total travel length of 3.15 m and 2.34 m respectively. On study day 2, the dual system allowed the identification of three different pendular types of motility at the first 10 min. These kinds were described as slow pendular (duration of 0–2.5 min, frequency of 1/20 ± 5 s), quiescence period (2.5–5 min) and a mixture of fast and slow pendular movements.

A different study used 10 mg of bisacodyl in order to create high amplitude propagated pressure waves (HAPPWs) in 10 healthy volunteers and assess them by manometry and cine-MRI at the same time over 24 min [24]. Overall, 11 HAPPWs were assessed by both techniques mostly 9–16 min following bisacodyl stimuli with a mean value of 63.5 mmHg. There was the case of three subjects were MRI identified three contractions which were not visible by manometry (‘negative contractions’). The authors suggested that the technique of cine-MRI is capable of bowel motility measurements and these measurements were in accordance with pressure changes assessed by the manometry.

The effect of ingestion of PEG electrolyte on the intestinal movements was studied in 24 healthy subjects [25]. They were divided into two equal groups who ingested a split dose of either 1 L the day before the MRI study day and the other 1 L on the MRI study day or a single dose of 2 L on the MRI scanning day. This study revealed that both dosing regimens had positive results but the single larger dose ascending colon movements caused twofold compared to the split dose (*p* = 0.0103). The positive changes in motion were correlated with ascending colon volumes (Spearman’s r = 0.53, *p* = 0.0128).

In a different study, a cine-MRI technique was developed for intestinal motility assessment and applied to four healthy subjects and eight possible IBD patients before and after intravenous injection of butylscopolamine [26]. In healthy volunteers, butylscopolamine reduced intestinal motion by (mean) 59% (*p* 1/4 = 0.171). Specifically, before the butylscopolamine injection, the mean score of the motility assessment in each volunteer was 910, 1605, 1860, and 5492 (mean 2467) and after the injection 524, 1057, 885, and 1557 (mean 1006). Mean difference was estimated to be 1461. Regarding the IBD patients, the investigators were able to identify the inflamed parts of the gut because of their reduced activity. The mean motility values of the maximum map were 15914, 4546, 5574, 9005, 8379, 8552, 7013, and 7637 and the respective ones of the mean map 5958, 1867, 2041, 3381, 3281, 3359, 2678, and 3385.

In another study, the impact of the laxative Moviprep on gut motion was assessed in 48 subjects (24 subjects with functional constipation or FC and 24 with IBS-C) [27]. The ascending colon motility index for subjects with FC was (mean (SD)) 0.055 (0.044) significantly lower than the one for IBS-C patients which was 0.107 (0.070) (line analysis 0.5 mm/s index), *p* < 0.01. After the Moviprep administration to the FC group, the required time for the first intestinal movement was (median (interquartile range)) 295 (116–526) min bigger than that for the IBS-C group which was 84 (49–111) min, *p* < 0.01. This first intestinal movement time was associated with the volume level in the ascending colon at that time (2 h) (higher volumes in the ascending colon caused delayed first bowel movements). Patients were considered to suffer from FC and not IBS-C when the required minutes for the first intestinal movement are above 230 (cut-off point, 55% sensitivity but 95% specificity). Considering the impact of Moviprep on intestinal movements, the FC group experienced a smaller intestinal activity for the first 24 h than the lower standard limit (6 movements) with the average number being 2–5 compared to the IBS-C average movement number which was 6–10, *p* < 0.01.

The motion of the ascending colon wall was also evaluated by MRI in 23 healthy subjects before and after a laxative stimulus [28]. The stimulus was provided by two different types of macrogol drinks. Eleven subjects ingested 1 L of macrogol whereas the remaining twelve drank 2 L. The team was able to identify the following five types of contractions—segmental antegrade, segmental retrograde, whole ascending colon antegrade and retrograde, and large amplitude contractions—and display them as a movie as they come from a cine database. The large amplitude contractions were simultaneous (<20 s on average) but affected the diameter of the whole gut. In most of the cases, these movements were followed by distension as well. A representative image of the motility assessment of this study is shown in Figure 1.

## 4. Colonic Chyme and Fluid

The appraisal of chyme characteristics and of water volumes in the colonic region has been a difficult task. This organ is poorly accessible in the physiological unprepared state but water distribution is a key determinant for oral drug absorption. Since standard MRI images are based on water hydrogen proton imaging, MRI could provide unique insights in the colonic undisturbed physiological chime and fluid state. MRI sequences typically used for cholangiopancreatography studies collect high signal from freely mobile fluids, which have long transverse relaxation time T2. T2 is one of the standard time constants of the MRI phenomenon and it is linked to water mobility. These MRI sequences are ideal to identify pockets of fluid in the body though signal from less fluid (‘thick’) components such as mucous are lost.

In a landmark study, the intestinal water distribution was estimated in 12 healthy subjects who consumed non-disintegrating capsules in fasting and fed conditions [29]. It was concluded that food ingestion had minimum effect on colonic water volumes (13 ± 12 mL) but increased the number of colonic pockets from 4 to 6 (*p* < 0.005). More specifically fasting inter-subject variability of colonic volumes was high (1–44 mL) and water pockets were mainly located in the cecum, ascending colon and descending colon with total capacity of (median) 2 mL. This variability remained high after the meal consumption too (2–97 mL) which caused increase in the number of water pockets (*p* < 0.005) but did not affect their volume capacity ((mean) 1 mL).

The intestinal freely mobile water content was also assessed in 18 healthy subjects who were administered mannitol as a model of secretory diarrhoea and capsules of placebo or loperamide or loperamide and simethicone at the same time [30]. MRI following the placebo intervention showed an increase in the water distribution in the ascending colon 45 min following the mannitol ingestion. On the contrary, at first (0–135 min) the other two active interventions caused the ascending colon water distribution to be reduced by more than 40% (*p* < 0.004). Later on (135–270 min), placebo behaved the same way as loperamide whereas, at the same time, loperamide combined with simethicone decreased the ascending colon water. MRI images of the placebo administration showed the presence of 6.9 ± 1.3 mL of water in the ascending colon similar to loperamide administration (6.8 ± 1.5 mL) and loperamide plus simethicone (4.5 ± 0.9 mL).

Additional MRI parameters that can be measured by MRI are the longitudinal relaxation time T1 and the transverse relaxation time T2. As already mentioned above, the relaxation times are fundamental time constants of the magnetic resonance environment of the water in the chyme, and are related to water mobility and the thickness of the colonic chime, whereby longer relaxation times generally relate to more liquid, less thick material. Use of the T2 relaxometry was explored in the mannitol/loperamide study mentioned above [30]. Loperamide and loperamide plus simethicone resulted in significantly lower T2 values than for placebo. Specifically, at a magnetic field of 1.5 T, at t = 90 min T2 values were 79 ± 4 ms, 67 ± 6 ms vs. 144 ± 28 ms (both *p* = 0.001) respectively and at t = 180 min loperamide plus simethicone resulted in T2 values of 130 ± 22 ms vs. 82 ± 6 ms for the placebo (*p* = 0.01). T1 and T2 relaxation time were also used in a study of ispaghula supplementation [31]. In the group of the healthy volunteers, again at a magnetic field of 1.5 T, T1 of the ascending colon was 720 (572–904) ms, 690 (594–911) ms, and 966 (67–1093) ms on maltodextrin, psyllium 10.5 g/d and psyllium 21 g/d intervention respectively. T1 of the descending colon was 440 (352–884) ms, 570 (473–700) ms, and 763 (575–985) ms respectively. T1 constant of the ascending colon of patients was 509 (472–670) ms and 890 (478–1030) ms on maltodextrin and on psyllium intervention while T1 of the descending colon was 213 (176–420) ms and 590 (446–1338) ms respectively. Respectively the T2 values in the ascending colon of the healthy group were 70 (56–72) ms, 73 (62–86) ms, and 83 (67–88) ms and T2 in the descending colon were 53 (40–67) ms, 54 (45–70) ms, and 74 (56–80) ms. The T2 of the patients’ ascending colon was 58 (42–73) ms on maltodextrin and 72 (51–105) ms on psyllium, while the respective values for the descending colon were 42 (34–52) ms and 66 (54–86) ms. Also, in a study of kiwifruit supplementation [32], the AUC (in seconds×minute units of measure) of the T1 constant of the ascending colon was 356 ± 109 on kiwifruit intervention and 291 ± 110 on control at time = 0–420 min whereas the AUC of the T1 from 240 to 420 min was 137 ± 39 and 108 ± 40, respectively. For the descending colon, the respective values were 216 ± 120 and 203 ± 114 from 0–420 min and 96 ± 50 and 87 ± 52 from 240–420 min. This initial body of work shows the absolutely unique ability of relaxation times measurements to detect changes in the properties of the colonic chyme in response to intervention without using invasive procedures.

**Figure 2:**
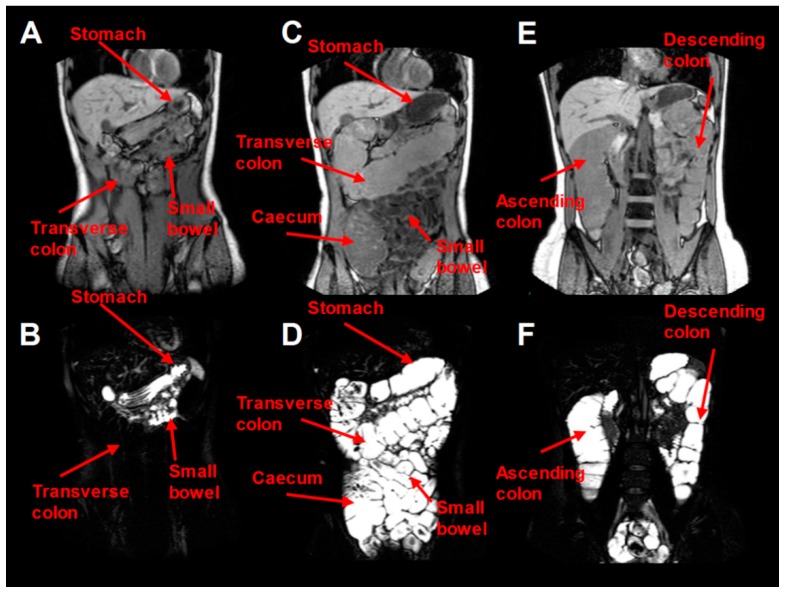
MRI quantification of water colonic content of one volunteer after ingestion of a single 2 L PEG electrolyte scheme. (**A**,**B**) concern fasting baseline conditions. White colour stands for water presence and black colour for water absence. In (**B**), water was found in the small bowel and the stomach (limited resting fluid) but not in the transverse colon. (**C**–**F**) conditions appear immediately after the ingestion of the 2 L PEG dose and therefore in (**D**) water was found in the stomach, the cecum and the transverse colon while (**F**) is a posterior image and represents water appearance in the ascending and descending colon. Reproduced from [25], Blackwell Publishing LTD, 2014 with permission.

The effect of per os PEG electrolyte in two dosing regimens (single of 2 L and split of 1 L twice) on colonic expansion was evaluated in 12 healthy volunteers [25]. They found out that the larger single dose had a higher positive impact in the total volume of the large intestine (102 ± 27%, range: 9–289%) than the split dose (35 ± 8%, range: 0–81%), *p* = 0.0332. The formulation affected mostly the ascending (single dose caused a greater increase, *p* = 0.0099) and the transverse colon rather than the descending colon (because of the defecation of the subjects the researchers assumed that it had passed through that region). The effect of the PEG dosing scheme on colonic water content is shown in Figure 2.

**Figure 3:**
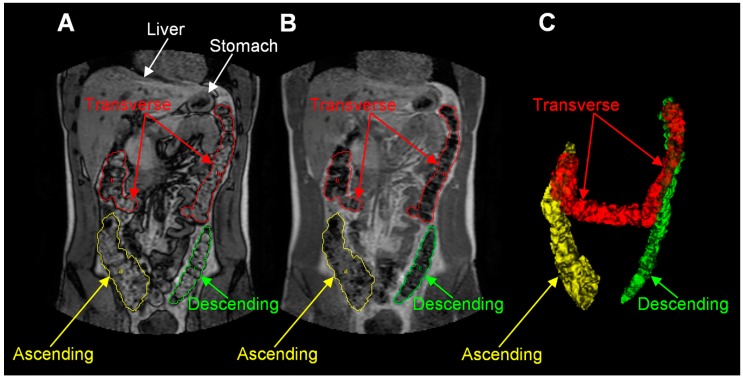
MRI images for colonic anatomy. (**A**,**B**) are different out of phase and in phase water and fat dual-echo imaging and (**C**) is a 3D reconstruction. Reproduced from [33], Blackwell Publishing LTD, 2013 with permission.

A different study quantified the gut segmental liquid presence in 25 IBS-D patients and 75 healthy volunteers in the fasted and in the fed (rice pudding) state [33]. The team found out that the fasted segmental volume in both groups was similar and specifically in healthy control group ascending colon volume was estimated 203 ± 75 mL, transverse colon volume 198 ± 79 mL and descending colon volume 160 ± 86 mL while from IBS-D patients 205 ± 69 mL, 232 ± 100 mL and 151 ± 71 mL respectively. On the contrary, food effect was not the same for the two study groups. As far as the healthy group is concerned, post feeding, through a higher ileo-colonic activity 10% expansion of the ascending colon content occurred and later (90–240 min) the last parts of the meal induced a smaller expansion in the same region. In the case of the IBS-D patients, feeding resulted in a smaller increase of the ascending colon volume and later (t = 90 min) a greater transverse one. A detailed MRI image of the colonic anatomy can be found in Figure 3.

The segmental and whole intestinal chyme content were studied in 25 healthy subjects [34]. This study also assessed variability between different study days. In addition, they also performed MRI in another seven healthy volunteers before and after defecation as they used stool volumes as a mean to validate the changes in colon volumes. Regarding the intestinal volumes on the two different scanning days, no significant overall or segmental change was detected (*p*>0.05) as overall volume was reported to be 760 mL (662–858) and 757 mL (649–865) on each observation. More specifically, on scanning day A the volume in the cecum/ascending colon was 177 mL (147–208) while on scanning day B 186 mL (159–212). In the same way, in the transverse colon it was estimated 192 mL (159–226) and 197 mL (155–240), in the descending colon 133 mL (110–157) and 193 mL (111–168) and in the rectosigmoid colon 257 mL (213–302) and 235 mL (193–277) respectively. The impact of defecation on regional volume distribution was only significant in the case of the rectosigmoid colon with 329 mL (248–409) before and 183 mL (130–236) after faecal excretion. In each other case, water volume in the cecum/ascending colon was 208 mL (167–248) and 198 mL (154–242), in the transverse colon 171 mL (100–242) and 173 mL (109–237) and finally, in the descending colon 185 mL (153–217) and 171 mL (129–212). Total intestinal water volume was reported 892 mL (723–1062) and 726 mL (635–816) prior and after defecation.

A different study of gut water quantification in 18 healthy volunteers focused on the effect of stress caused by IV administration of corticotrophin releasing hormone (CRH) and cold water hand immersion on the intestinal environment [35]. The volunteers were divided in two groups and were fed a standard test meal (rice pudding). After the comparison of the saline and the CRH arms of the study, it was found that CRH injection expanded the intestinal water presence from (mean AUC (SD)) 46,227 (10,927) to 49,817 (10,770), ANOVA *p* = 0.002. Comparison of ice water immersion to warm water immersion revealed no differences with the corresponding values being 48,991 (17,501) and 48,964 (16,950) respectively, *p* = 0.730. The consumption of the meal did not increase significantly the ascending colon water volume (t = 0–45 min) only on CRH injection (15 (32) mL, *p* = 0.3). On saline injection, warm immersion and ice immersion water volume increased significantly (16 (32) mL, *p* = 0.040, 33 (51) mL, *p* = 0.020, 22 (27) mL, *p* = 0.005 respectively). Transverse water volumes were only significantly affected (decreased) only on water immersion (*p* = 0.0107). Water distribution in the descending colon was unaffected in any case.

The regional colonic water was studied in four healthy subjects finding that the median (interquartile range) of total colon volume was 819 mL (687–898.5). Regarding each segment, the volume of the ascending colon was determined as 200 mL (169.5–260), the volume of the transverse 200.5 mL (113.5–242.5), the descending 148 mL (121.5–178.5) and in the final part of the colon (sigmoid-rectum) 277 mL (192–345) [36].

A different study investigated how gluten affects the colonic volume and gas in 12 healthy subjects [37]. Fasted colonic volume in total after gluten-free bread (GFB) diet was (mean ± SEM) 748 ± 258 mL, after normal gluten content bread (NGCB) 659 ± 291 mL and after added gluten content bread (AGCB) 576 ± 252 mL. Segmental respective volumes were estimated as following: ascending colon 250 ± 119 mL, 256 ± 149 mL, 224 ± 128 mL, transverse colon 289 ± 95 mL, 212 ± 73 mL, 178 ± 86 mL, and descending colon 209 ± 73 mL,187 ± 92 mL, and 172 ± 77 mL. Statistical differences were only detected in the case of the volume of the transverse colon which was higher after the GFB diet compared to NGCB and AGCB diets (*p* = 0.02).

The effect of a laxative PEG electrolyte formulation on gut volumes was studied in 24 patients with functional constipation and 24 with IBS-C [27]. Baseline measurements in the fasted state revealed higher ascending colon content in the FC group (mean (SD) 314 (101) mL) when compared to the IBS-C group (226 (71) mL, *p* < 0.001). The same applied to the overall colonic volumes which were 847 (280) mL and 662 (240) mL, respectively (*p* = 0.03). Similar differences existed in the volumes of the ascending colon 120 min after the PEG electrolyte ingestion which were 597 (170) mL and 389 (169) mL respectively (*p* < 0.01) and the total large intestinal volumes 1505 (387) mL and 1039 (418) mL respectively (*p* < 0.01) at the same time.

The impact of the co-administration of fructose and corticotropin-releasing factor (CRF) or saline on the intestinal water was studied in a healthy group of 11 male and 10 female volunteers [38]. On the CRF arm of the study, the baseline of the ascending colon water volume was 210 ± 77 mL (t = −45 min) and after the administration of the fructose meal the volume rose to 270 ± 109 mL. This increase was more intense (29%) compared to the saline effect (12% rise) where the volume was measured 226 ± 74 mL and 252 ± 83 mL respectively. The study showed higher ascending colon water in men than in women only in the case of CRF administration.

The effect of oxycodone administration on the regional gut water distribution of healthy subjects was also characterised [39]. The researchers evaluated the segmental intestinal volumes in two arms (oxycodone and placebo) and in two different days (day 1 and day 5). On oxycodone, there was a volume rise in caecum/ascending colon mean (95% confidence interval) from day 1 (177 (147–208) mL) to day 5 (249 (209–291) mL), *p* = 0.005 (statistically significant), in transverse colon from 192 (159–226) mL to 230 (190–270) mL, *p* = 0.005 (statistically significant) respectively and in descending colon from 133 (110–157) mL to 153 (132–175) mL (*p* = 0.08) respectively. On the contrary, there was a non-significant decrease in the rectosigmoid colonic volume from 257 (213–302) mL to 249 (213–284) mL respectively (*p* = 0.64) but overall oxycodone affected positively the total large intestinal volume (760 (662–858) mL to 881 (783–979) mL respectively, *p* = 0.008 (statistically significant)). The placebo ingestion lead to a rise in the caecum/ascending colon volume from 186 (159–212) mL in day 1 to 211 (184–238) mL in day 5 (*p* = 0.03, statistically significant) and in rectosigmoid colon volume from 235 (193–277) mL to 244 (200–288) mL respectively (*p* = 0.06). At the same time there was a decrease both in transverse colon volume from 197 (155–240) mL to 183 (152–213) mL respectively (*p* = 0.57) and in descending colon volume from 139 (111–168) mL to 121 (101–142) mL respectively (*p* = 0.07). Overall, the placebo administration scheme did not affect significantly the total large intestinal volume (757 (649–856) mL to 759 (670–848) mL respectively, *p* = 0.26).

The intestinal liquid and non-liquid content was also evaluated in 10 healthy volunteers in regards with high- and low-residue diets, meals and faecal output [40]. Low-residue diet affected positively the non-gaseous content in the right region of the colon which climbed up to 41 ± 11 mL 4 h following the meal ingestion compared to fasted state (−15 ± 8 mL; *p* = 0.006 vs. fed). A significant reduction on the non-gaseous content caused by the faecal output was only present in the distal intestine. As far as the meal effect on the colonic contents is examined, non-gaseous colonic content had escalated only in the proximal intestine (by 37% ± 14%, *p* = 0.007) in the case where subjects underwent a fasting scan and a second scan 4 h following the meal ingestion. Generally, the gas content had risen (by 31% ± 14%, *p* = 0.064). In the fasted state, there was no reduction in the non-gaseous content 4 h after the first scan (−29% ± 11%, *p* = 0.040). The administration of the meal (fed state) had a statistically significant effect (increase) only in the volume of chyme excluding gas (*p* = 0.006 vs. fed). Faecal output lowered the gas volume as well as the non-gaseous (*p* < 0.001) in the whole intestine and the biggest change was in the pelvic colon. This output consisted the 38% ± 5% of the chyme, excluding gas.

The segmental water volumes were also measured as a part of a study of 34 healthy subjects and 30 patients with IBS-D, 16 with IBS-C and 11 with IBS-M in the fasted and the fed state (using a rice pudding, strawberry jam, wheat bran, and orange juice test meal) [41]. At t = 45 min, in the fasted state, colonic volume of the ascending solon was estimated (median (interquartile range)) 194 (150–234) mL in the healthy group, 217 (191–268) mL in the IBS-C group and 209 (147–248) mL in the non-constipated IBS or IBS-nonC group respectively. Respectively, the transverse colonic volume was 165 (117–255) mL, 253 (200–329) mL, and 198 (106–248) mL and the reduced volumes in the IBS-C group were the only ones to be considered as statistically significant (*p* = 0.02). For the descending colon, the respective values were (mean (SD)) 143 (61) mL, 153 (47) mL and 114 (52) mL when the overall intestinal volume was found 513 (174) mL, 644 (148) mL, and 498 (175) mL respectively. Postprandially, at t = 0, the volumes in the ascending colon increased slightly in every group apart from the IBS-D group but not significantly and then decreased in all groups. When transverse colon is concerned, volume were unchanged at first but increased at t = 180 min reaching a significant high state at t = 405 min (compared to the healthy group, *p* = 0.04) but generally unaffected compared to fasting transverse volumes at t = −45 min of IBS-C and IBS-nonC group. Overall, the intestinal volumes were higher in IBS-C than in IBS-nonC.

The effect of consuming glucose, fructose and inulin on intestinal water volumes was evaluated in 29 healthy volunteers and 29 IBS patients [42]. In the healthy group, glucose ingestion caused a reduction in the total colonic volume (mean (±SEM)) of 20.9 (15.7) mL whereas fructose and inulin increased it about 50.8 (16.2) mL and 136.8 (17.6) mL on average respectively. Regarding the patients’ group, glucose affected the gut volume the least causing a slight rise of 3.8 (11.4) mL while fructose and inulin caused a bigger rise of 58.5 (20.3) mL and 129.6 (19.9) mL respectively. The researchers concluded that intestinal volume was primarily affected by fructose and inulin rather than glucose in both groups.

**Figure 4:**
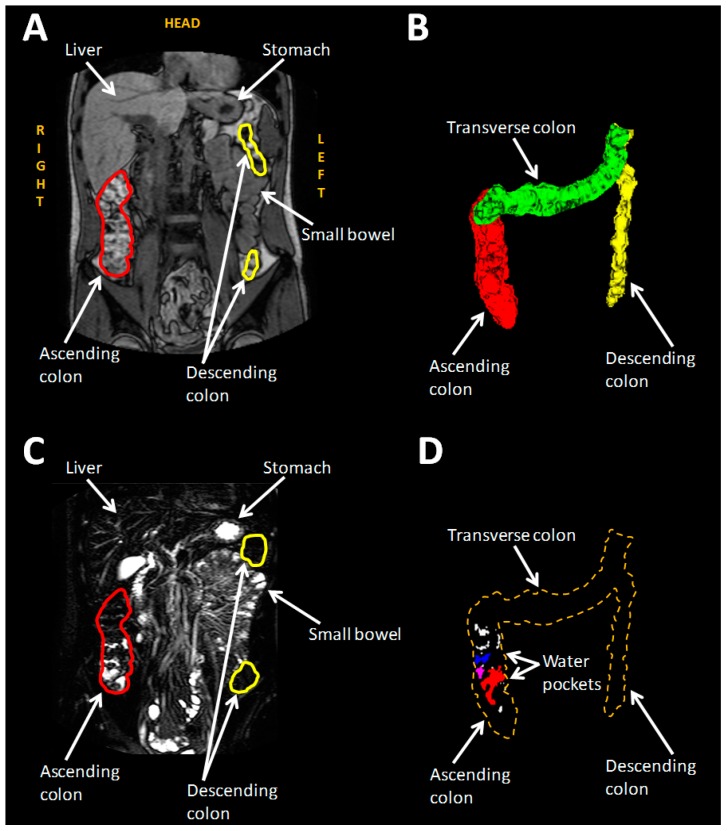
(**A**) MRI bowel image. (**B**) 3D reconstruction of the bowel. Red, green, and yellow stand for ascending, transverse and descending colon respectively. (**C**) Highly T2 weighted image with the same angle of (**A**). Unbound liquid appears white whereas bound and less mobile liquid seem darker. (**D**) Unbound liquid of the colon. Reproduced from [43], American Chemical Society, 2017 with permission.

A different study estimated the timeline of total and segmental unbound water in terms of volume and number of liquid pockets (Figure 4) in the large intestine of 12 healthy volunteers after the administration of 240 mL of water being the first team to conduct this kind of study [43]. They found that the number of bound colonic water pockets water in the fasted state were 11 ± 5 mL and each of them contained approximately 2 ± 1 mL in total. 30 min after the water ingestion the colonic liquid reached the peak of 7 ± 4 mL divided in 17 ± 7 pockets. By time, the number of the liquid pockets and their volumes decreased but, one hour later, only the amount of the colonic pockets peaked again (17 ± 7) while their volumes remained in lower levels (3–4 mL). The team reported that there was a high variability regarding the number of the pockets (0 to 89) and the total unbound colonic water (0 to 49 mL). They also reported that the main site of the freely liquid was the ascending colon.

The colonic water distribution was also evaluated as part of a study of the effect of macrogol on large bowel flow of 11 healthy and 11 constipated volunteers [44]. Baseline scanning revealed very little water content and no significant differences between the healthy and the constipated subjects (2 (0–7) mL and 11 (1–29) mL respectively, *p* = 0.16). At t = 60 min, macrogol boosted significantly the water volumes in both healthy (140 (104–347) mL, *p* = 0.001) and constipated (228 (91–259) mL, *p* = 0.0039, (Wilcoxon ranked pairs test)). 120 min following the macrogol administration, the ascending colon unbound water content was still significantly high in both groups (healthy: *p* = 0.002 and constipated: *p* = 0.0039) but it had decreased in the patient group where it was found 84 (3–195) mL compared to control group [146 (32–227) mL].

The regional and total bowel water was assessed in 9 healthy subjects and 20 constipated patients on maltodextrin (placebo), 10.5 g and 21 g of psyllium [31]. In both groups, in the fasting state colonic volumes were risen from (median (interquartile range)) 372 (284–601) mL to 578 (510–882) mL in healthy controls and from 831 (745–934) mL to 1104 (847–1316) mL in patients by the administration of 7 g of psyllium (*p* < 0.05). The ascending colon water was (median (interquartile range)) 0.2 (0.1–0.6) mL in the maltodextrin intervention, 4.0 (2.4–7.0) mL and 7.4 (2.8–16.5) mL in the 10.5 g/d and 21 g/day psyllium intervention respectively in the healthy group whereas in patients group it was estimated 0.13 (0.01–0.66) mL on maltodextrin administration and 3.41 (0.10–7.69) mL on 21 g/day psyllium administration. Segmentally, significant differences occurred while on the placebo intervention in the colonic volumes of the fasted state between patients and healthy subjects (745 (455–844) mL and 372 (284–601) mL respectively, *p* < 0.5). The cause of these differences were mainly because of higher volumes in the ascending and transverse region of the intestine.

The effect of oxycodone plus macrogol and prolonged-release (PR) naloxone and oxycodone on the segmental gut water of 20 healthy subjects was evaluated [45]. The comparison of baseline and day 5 scanning of PR naloxone intervention showed that regional intestinal volumes increased in the cecum/ascending colonic volume (mean (±SD)) from 220 ± 25 mL to 257 ± 41 mL (*p* = 0.156), the transverse colonic volume from 258 ± 42 mL to 295 ± 47 mL (*p* = 0.161), the descending colonic volume from 187 ± 32 mL to 210 ± 51 mL (*p* = 0.384) and the total from 941 ± 108 mL to 1036 ± 176 mL (*p* = 0.087) respectively. The volume also increased from 276 ± 60 mL to 273 ± 71 mL (*p* = 0.904) in the rectosigmoid colon. The same comparison of the macrogol intervention caused a volume rise in each intestinal region and more specifically in the ascending colon where volumes rose from 216 ± 39 mL to 277 ± 53 mL (*p* = 0.005), in the transverse colon from 270 ± 59 mL to 328 ± 51 mL (*p* = 0.006), in the descending colon from 184 ± 55 mL to 231 ± 44 mL (*p* = 0.022), in the rectosigmoid colon from 242 ± 55 mL to 287 ± 52 mL (*p* = 0.026) and in total from 912 ± 158 mL to 1123 ± 145 mL (*p* < 0.001). The results showed that the macrogol administration increased the volume significantly in every segment. Significant volume rise in the whole intestine occurred only on macrogol administration.

The role of a low fermentable oligo-, di-, mono-saccharides and polyols or FODMAP regimen with the administration of either oligofructose or maltodextrin on the intestinal water volumes was investigated in 37 healthy volunteers [46]. MRI analysis revealed that both oligofructose and maltodextrin exerted a positive effect on the intestinal volume since it was increased on average from 110 mL, 95% CI 30 mL to 190 mL, *p* = 0.01 (19.6% mean increase) and from 90 mL, 95% CI 6 mL to 175 mL, *p* = 0.04 (15.5% mean increase) respectively. When it comes to segmental colonic volumes only oligofructose was capable to rise the volumes’ values in the ascending (mean 35 mL, 95% CI 9 mL to 61 mL, *p* = 0.01), transverse (mean 44 mL, 95% CI 4 mL to 84 mL *p* = 0.03) and distal colon (mean 26 mL, 95% CI 0 mL to 52 mL, *p* = 0.05). It was also noted that there was no significant difference when the results from the two substances were compared to each other.

The role of foods in modulating gut water content of 15 subjects and either a bread meal, a rhubarb meal, or a lettuce meal [47]. The bread meal created an AUC (area under the curve) (mean (SEM)) of 78 (43) mL whereas the rhubarb and the lettuce meal created a much higher rise of 291 (89) mL (*p* < 0.01 Wilcoxon) and 409 (231) mL respectively.

## 5. Colon Transit and Luminal Flow

Transit times of oral dosage forms exert a significant role on their efficacy. Modified release oral formulations and colon-targeted drugs have to release their active substance in the colon. Therefore, changes in the transit and flow patterns may affect drug release. MRI has been increasingly used to study the transit times as summarised in Table 1.

Tagging is a MRI method commonly used to assess motion in the heart in cardiac imaging. It has been used previously in the stomach environment and is now finding new applications to assess colonic luminal motion of the chyme [44]. Pritchard et al. evaluated the application of MRI tagging to study the ascending colonic flow in 11 healthy and 11 constipated volunteers on magrocol administration. Analysis of the baseline scanning revealed weak flow procedures regardless volunteer group (healthy 20% (14–23), constipated 12% (11–20), *p* = 0.1). 60 min after macrogol administration, dislocation of the tags could be observed mainly in the healthy group (30% (26–35), *p* = 0.002, Wilcoxon test) rather than the constipated one (17% (13–230, *p* = 0.57, Wilcoxon test). This dislocation was characterized as forward and backward and took place at the same time and region of the ascending colon (central). Moreover, there was a fast (>4.8 cm) retrograde central ‘jet’ and a decrease in the tag intensity as well. Results from all the scanning allowed the observation of higher central motion in the ascending colon rather membrane motion which was weaker but still detectable at t = 120 min mainly in the healthy large intestine (25% (18–360), *p* = 0.002) and not in so much in the constipated (13% (12–180), *p* = 0.76). The researchers concluded that there were significant differences in the %COV in each time point between the two conditions (60 min (*p* = 0.0020), 120 min (*p* = 0.003), Mann–Whitney rank sum test).

## 6. Conclusions and Future Outlook

Recent developments in MRI imaging techniques have opened up the possibility to expand knowledge of the colonic environment providing unprecedented insights on colonic dimensions, chyme and fluid characteristics, motility, transit, and flow. These physiological parameters will, in turn, have profound impact on drug dissolution and absorption. New knowledge of colonic parameters can also increase the in vivo relevance of in vitro models. Colonic motility has yet to be fully included in the biorelevant in vitro modelling [43]. MRI has the potential to become the modality of choice for early phase assessment of new colonic-targeted drugs’ functionality confirming their proof of concept and helping to explain mode of action [11,31]. This is of great regulatory value. The future of MRI of the abdomen includes ultra-high-field (7 T and above) scanners [53] and developments in the design of open magnets that have the advantage of imaging volunteers in the sitting position [11,54]. Also, susceptibility differences and air-tissue interfaces can be responsible for generating artifacts, some of which may be difficult to remove though some could potentially be eliminated by using diamagnetic elements to improve the quality and reliability of the images and data acquired [55]. Another great advantage of MRI is its non-ionizing nature [56]. This can help to extend the studies of gut physiology to children, a group where knowledge of the physiological GI parameters is particularly scanty. Furthermore, one of the latest advancements in the MRI field is the use of ultrashort echo time (UTE) MRI, which has been applied for abdominal imaging purposes [57,58]. The UTE abdominal MRI has not been established and standardized yet but is has great potential since it allows imaging areas whose signal decay fast [58]. Lastly, another common method to minimize abdominal motion artifacts is the application of respiratory gating techniques and this practice could reduce the needs for breath holds or allow for longer sequences [55].

However, there exist some limitations regarding the imaging of the colon and regional drug behaviour. The colon is a large organ and current imaging of the whole colon at once is not of high temporal quality and therefore development of 4D MRI imaging could be helpful [9]. Modified release solid formulations deliver the active substance 6–24 h after administration and for this reason MRI should be applied on the large intestine for longer than the usual 2 h [43]. The bore of the magnet may feel small to claustrophobic participants and can limit studies in clinically obese subjects [11]. In the case of very young children, sedation might be suggested [55]. When it comes to reduction of motion artifacts and bowel movement, antiperistaltic drugs and gating correction techniques could be of use to reduce artifacts [55]. MRI cannot provide luminal pressure data which are of interest too. Specifically, motility is not periodical with large time intervals of relative quiescence between larger contractions [9]. Further studies are needed to analyse the potential of MRI for the exploration of the interactions between the mixing and transportation of contents with meal type. Analysis of flow types such as antegrade and retrograde can reveal the ascending colon’s ability of mixing and propelling its contents. Breath hold imaging techniques can help to remove respiratory artifacts and develop MRI as a tool [44]. Furthermore, carrying out MRI experiments is still perceived as expensive compared to well-established techniques in the pharmaceutical field such as gamma-scintigraphy when, on the same time, it yet needs to be optimized and validated towards them [11,31], though costs are decreasing. A comprehensive MRI study day provides a large amount of data, which requires extended image processing time. Practices vary between labs and therefore there is increasing need of standardisation and image analysis automation [11].

In conclusion, MRI of the colon has developed substantially over the recent period considered in this review, and further exciting developments can be expected. The novel physiological insight will no doubt have a profound impact on our understanding of the colonic environment for drug delivery and absorption and will ultimately help to increase the in vitro/in vivo relevance of computer simulations and bench models.

## Figures and Tables

**Table 1:** Colonic transit of contents with various MRI techniques.

Reference	Aims	Methods	Outcomes
[29]	Assessment of the intestinal transit by MRI	12 healthy volunteers were scanned in fasted and fed state and after consumption of gel-filled capsules	Location of the capsules was affected by food consumption (in the large intestine: fasted vs. fed state was 3 vs. 17 capsules respectively, *p* < 0.01)
[48]	Assessment of new MRI technique of estimating intestinal transit with per os capsules containing gadolinium-saline solution	7 females and 8 males (all healthy) consumed 5 capsules	Mean transit time for female and male volunteers was 41 ± 9 h and 31 ± 10 h respectively
[23]	Application of ^19^F and ^1^H MRI on intestinal transit	2 healthy subjects consumed perfluoro-[15]-crown-5-ether capsules: 1 each on scanning day 1 and 2 each on scanning day 2	Single capsule tracking: total transit lasted 27 h and 32 h for subjects A and B respectively (mean capsule velocity was 1.0 mm/s and 1.0 mm/s respectively). Capsule found outside the stomach 170 min and 220 min respectively Dual capsule tracking: capsules located out of the stomach 210 min after ingestion
[49]	Validate MRI technique towards OCTT^3^ and WGT^1^ measurements	21 healthy subjects OCTT^3^ estimated by the arrival of the head of the meal into the beginning of the large bowel with MRI and by LUBT^4^ WGT^1^ estimated by MRI marker capsules and ROMs^5^	MRI measurement of OCTT^3^ was (median(IQR)) 225 (180–270) min and of WGT^1^ was 28 (4–50) h
[25]	Investigation of the effect of oral PEG electrolyte in two dosing regimens on colonic motility	12 healthy subjects consumed the split dose (1 L before the first scanning day and 1 L on the scanning day) and the other 12 healthy volunteers the single dose (2 L on the first scanning day) Each volunteer ingested MRI marker pills the day before the MRI transit scan (days 8, 14, 28)	No differences due to dosing regimens as Mean position score of split vs. single dose at Day 8: 6.2 ± 0.4 vs. 5.4 ± 0.6, *p* = 0.2527, Day 14: 5.8 ± 0.4 vs. 5.5 ± 0.5, *p* = 0.6076, Day 28: 6.1 ± 0.5 vs. 6.6 ± 0.3, *p* = 0.3327 No differences between the days regardless dosing: Day 8 vs. 14: *p* = 0.7750 Day 8 vs. 28: *p* = 0.2350
[50]	Evaluation of MRI techniques of OCTT^3^ assessment towards LHBT^6^ in healthy volunteers	28 healthy volunteers were recruited OCTT^3^ was assessed by the arrival of the head of the lactulose ingestion (10 g/125 mL)	OCTT^3^ by MRI measurements was (median (IQR)) 135 (120–150) min
[27]	MRI investigation of the effect of PEG electrolyte as a laxative on the colonic environment	24 patients with functional constipation and 24 with IBS-C participated in this study. They has to consume 5 MRI marker pills before the scanning day and 1 L of PEG electrolyte after the baseline scan on the study day	WAPS^2^ for FC (3.6 (2.5–4.2)) was higher than the IBS-C (2.0 (1.5–3.2)), *p* = 0.01
[41]	Distinguish subgroups of IBS based on MRI markers	91 volunteers took part (34 healthy, 30 with IBS-D, 16 with IBS-C, and 11 IBS-M as mixed. IBS-M and IBS-D were listed as IBS-nonC)	WGT^1^ for IBS-C, healthy volunteers and IBS-D was 69 (51–111) h, 34 (4–63) h and 34 (17–78) h respectively and OCTT^3^ was 203 (154–266) min, 188 (135–262) min and 165 (116–244) min respectively
[44]	Study the ascending colonic transit in healthy and constipated subjects	11 healthy and 11 constipated subjects were scanned fasted and after ingestion of 500 mL of macrogol and consumption MR markers	WAPS^2^ between healthy and patients was (median (IQR)) 0.6 (0–1) and 2.6 (1.4–3.6) respectively, *p* = 0.0011
[51]	Evaluation of the applicability of gadolinium filled MRI capsules towards radio-opaque markers (ROMs^5^) on colon transit time (CTT)	7 constipated and 9 healthy subjects ingested 5 gadolinium-based capsules as MRI markers and 20 ROMs^5^	MRI measurements revealed that CTTs in healthy and constipated were 30.9 ± 15.9 h and 74.1 ± 7.2 h respectively, *p* < 0.05 Patients had higher CTTs than the healthy ones
[52]	Establishment of an MRI technique for bowel motion and transit assessment	Baseline and fed state MRI scanning of 15 healthy subjects Meal: chicken or mushroom soup Each subject consumed 5 MRI capsules of Gadoteric acid the day before the study day	WAPS^2^ (24 h) = 1.0 (0–3.8) WGT^1^ (hours) = 33 hr
[31]	Evaluation of psyllium consumption on colonic environment of healthy and constipated volunteers	9 healthy subjects received maltodextrin (placebo) and psyllium 10.5 g and 21 g for 6 days randomly and 20 constipated subjects ingested maltodextrin and 21 g of psyllium in the same way On treatment day 5, each volunteer ingested 5 MRI marker capsules with gadoteric acid	WGT^1^ was higher in healthy than patients (*p* < 0.05) Controls: WAPS^2^24 showed no differences as (median (IQR)) it was 1.0 (0.1–2.2) on maltodextrin, 1.4 (0.2–2.1) on 10.5 g of psyllium and 0.6 (0–1.9) on 21 g of psyllium Patients decreased from 4.2 (3.2–5.3) on maltodextrin to 2.0 (1.5–4.0) on psyllium (*p* = 0.067)
[32]	Evaluation of intestinal volumes and function on kiwifruit consumption	2 kiwifruits or maltodextrin (control) 2 times per day for 3 days in the fasted and fed state	WGT for kiwifruit was (median (IQR)) 0.8 (0–1.4) and for control 1.0 (0.5–3.1), *p* = 0.11

WGT^1^: whole gut transit WAPS^2^: median average weighted position score OCTT^3^: orocecal transit time LUBT^4^: lactose ureide breath test ROMs^5^: radio-opaque markers LHBT^6^: lactulose hydrogen breath test.
